# High *SLC4A11* expression is an independent predictor for poor overall survival in grade 3/4 serous ovarian cancer

**DOI:** 10.1371/journal.pone.0187385

**Published:** 2017-11-01

**Authors:** Lianzhi Qin, Ting Li, Yuhua Liu

**Affiliations:** 1 Delivery room, Linyi Central Hospital, Linyi, Shandong, China; 2 Department of Gynecology, Zoucheng People’s Hospital, Zoucheng, Shandong, China; 3 Department of Obstetrics, Anqiu People’s Hospital, Anqiu, Shandong, China; Sapporo Ika Daigaku, JAPAN

## Abstract

In this study, we aimed to examine the expression of *SLC4A11* in ovarian cancer and in normal ovarian tissues, its prognostic value and the possible mechanism of its dysregulation. Bioinformatic analysis was performed by using data from the GEO datasets, the Cancer Genome Atlas-Ovarian Cancer (TCGA-OV) and the Human Protein Atlas (HPA). Results showed that *SLC4A11* was upregulated in ovarian cancer compared with normal ovarian epithelial tissues. In patients with primary serous ovarian cancer in TCGA-OV, the cases with lymphatic invasion (N = 133) had significantly higher *SLC4A11* expression than those without lymphatic invasion (N = 77) (*p* = 0.0069). High *SLC4A11* expression was consistently associated with worse overall survival (OS). Univariate and multivariate analysis confirmed that high *SLC4A11* expression was an independent prognostic factor for poor OS in grade 3/4 (G3/G4) tumors (HR = 1.416, 95%CI: 1.098–1.824, *p* = 0.007). 320 out of 578 (55.4%) ovarian cancer cases had SLC4A11 amplification. High methylation group had a significantly lower level of *SLC4A11* expression. Based on these findings, we infer that high *SLC4A11* expression is an independent predictor for poor OS in grade 3/4 serous ovarian cancer. Both DNA amplification and hypomethylation contribute to its upregulation in ovarian cancer.

## Introduction

Intracellular and extracellular pH (pH_i_ and pH_e_) homeostasis is an important constitution of cellular microenvironment and is a prerequisite for normal cell function [1]. Studies in the past decade revealed that pHi homeostasis is often dramatically altered in cancer [2, 3]. Cancer cells usually keep a pHi that is equal to or even more alkaline than the surrounding normal counterparts, suggesting that they upregulate net acid extrusion [2, 3]. This alteration leads to at least two fundamental differences to normal physiology: firstly, it is supportive to maintain pHi homeostasis by eliminating excessive production of acid equivalents in cancer cells due to hypoxia and/or oncogene-induced changes in glycolytic metabolism [4]; secondly, prolonged extracellular acidification (pH_e_), which favors tumor invasion and metastasis [5, 6].

Solute linked cotransporter 4 (SLC4) family is comprised of ten members (SLC4A1-5; SLC4A7-11), which have critical roles in pHi buffering [7, 8]. SLC4A1-3 are Cl^−^/HCO_3_^−^ exchangers and function as cellular acid loaders [9]. SLC4A4, −4A5, −4A7, −4A8 and −4A10 are Na^+^-coupled HCO_3_^−^ transporters and function as cellular acid extruders [9]. SLC4A11 is the most divergent member of this family and has recently been characterized as a Na^+^/OH^−^ and NH4^+^ transporter [10]. Therefore, SLC4A11 can mediate H^+^ efflux and can be considered as a cellular acid extruder. Dysregulated SLC4 family members have been implied in pathological development of some cancers. For example, SLC4A7 regulates pHi and tumor cell progression in breast cancers [11, 12]. Upregulated *SLC4A4* contributes to growth and migration of colon and breast cancer cells [13]. *SLC4A9* disruption by either genetic or pharmaceutical approaches results in pHi acidification and reduced cell growth of breast cancer and glioma cells [14].

Ovarian cancer cells have an elevated H^+^ efflux compared with non-tumor cells [15]. One recent study found that the basal pH_i_ is higher in ovarian cancer A2780 cells than in normal ovarian HOSE cells [16]. *SLC9A1* amplification is a mechanism of the high basal PH_i_ and is associated with unfavorable overall patient survival [16]. These findings suggest that pHi regulation is closely related to ovarian cancer cell behaviors and prognosis of the patients.

In this study, we examined the expression of *SLC4A11* in ovarian cancer/normal ovarian tissues and further assessed its prognostic value and the possible mechanism of its dysregulation.

## Materials and methods

### Bioinformatic data mining in GEO

The normalized data of one previous microarray (GDS3592) [17] that explored the dysregulated genes in ovarian cancer epithelial cells (CEPIs) from patients with primary serous ovarian cancer compared with normal ovarian surface epithelia (OSE) was downloaded from GEO dataset for secondary analysis.

### Data mining in the Human Protein Atlas

*SLC4A11* expression at the protein level in normal ovarian tissues and in serous ovarian cancer tissues was compared by using the immunohistochemistry (IHC) staining data provided by the Human Protein Atlas (HPA) (http://www.proteinatlas.org/) [18, 19].

### Bioinformatic analysis using data from TCGA-Ovarian Cancer (TCGA-OV)

*SLC4A11* expression, its copy number alteration and its DNA methylation in patients with ovarian cancer were examined using data from TCGA-OV. Original data downloaded from the UCSC Xena Browser (https://xenabrowser.net/) were given in S1 Table. The association between *SLC4A11* expression and overall survival (OS) or recurrence-free survival (RFS) was assessed by generating Kaplan-Meier survival curves, with median *SLC4A11* expression as the cutoff. The analysis was performed using the UCSC Xena Browser or by using GraphPad Prism 6.0. Among 595 patients with primary serous ovarian cancer in TCGA-OV, 540 patients had *SLC4A11* expression measured by AgilentG4502A_07_3. This proportion of patients was included in the univariate and multivariate analysis of the association between *SLC4A11* expression and OS/RFS.

### Statistical analysis

The association between clinicopathological characteristics and *SLC4A11 e*xpression was assessed by using χ^2^ tests. The significance of the difference between the survival curves was assessed by log-rank test. Univariate and multivariate Cox regression models were used to evaluate prognostic significance. Welch’s t-test was conducted to compare *SLC4A11* expression between patients with or without lymphatic invasion and between groups with high/low methylation. *p*<0.05 was considered statistically significant.

## Results

### *SLC4A11* is upregulated in ovarian cancer compared with normal ovarian epithelial tissues

**Fig 1:**
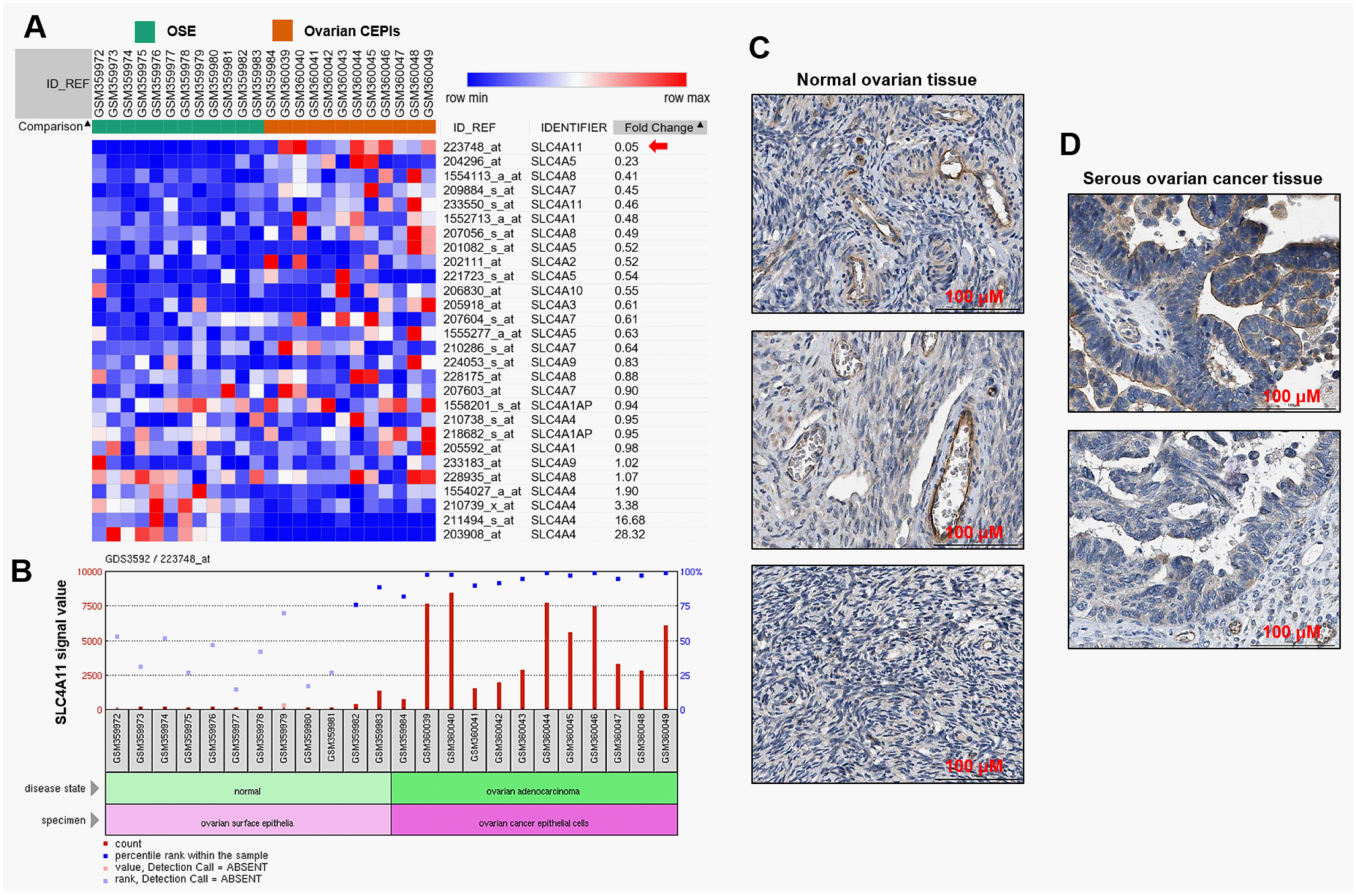
*SLC4A11* is upregulated in ovarian cancer compared with normal ovarian epithelial tissues. **A**. Heat map of the expression of SLC4A family members in 12 cases of ovarian CEPIs compared with 12 cases of OSE. Red: up-regulation; Blue: down-regulation. The image was generated by re-analysis of the raw microarray data of GDS3592. **B**. *SLC4A11* microarray signal values in 12 CEPIs and 12 OSE cases. Data were analyzed by using the tool provided by GEO datasets. **C-D**. Representative images of IHC staining of SLC4A11 in normal ovarian tissues (C) and serous ovarian cancer tissues (D). Data were obtained from the HPA: http://www.proteinatlas.org/ENSG00000088836-SLC4A11/pathology/tissue/ovarian+cancer.

By re-analysis of the normalized data of GDS3592, we examined the expression of SLC4A family members in CEPIs and OSE (Fig 1A). *SLC4A11* was the most significantly upregulated gene among the 10 SLC4A family members (Fig 1A, red arrow). The bar chart of the array signaling value further indicated that *SLC4A11* was significantly upregulated in the CEPI samples compared with the OSE samples (Fig 1B). By data mining in the HPA, we found that in normal ovarian tissues, follicle cells had medium SLC4A11 staining (Fig 1C). But SLC4A11 expression was not detectable in ovarian stroma cells (Fig 1C). In comparison, the serous ovarian cancer tissues usually had low to medium SLC4A11 staining in both cytoplasma and cell membrane (Fig 1D).

### High *SLC4A11* expression is associated with lymphatic invasion

**Fig 2:**
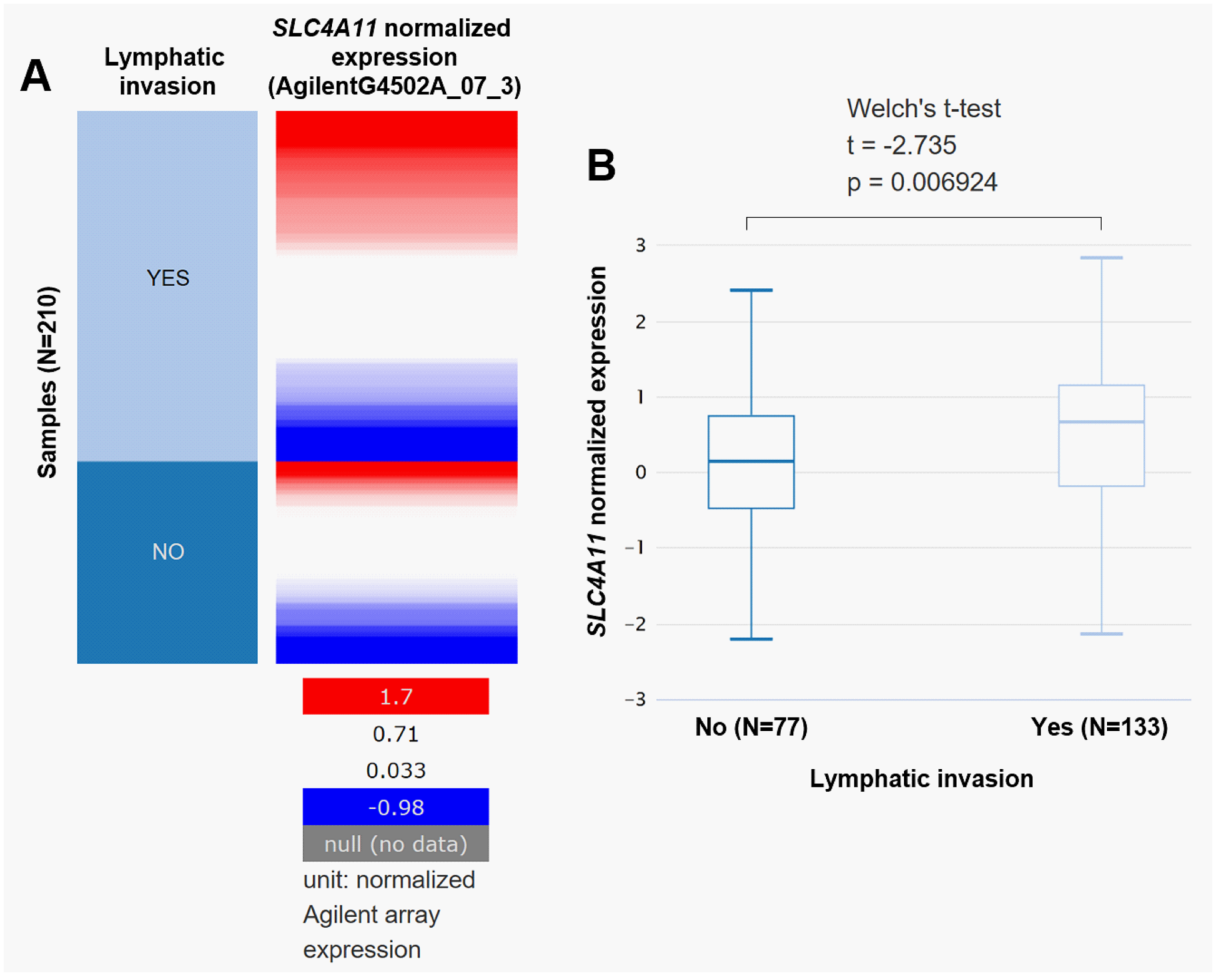
High *SLC4A11* expression is associated with lymphatic invasion. **A-B**. Heat map (A) and box plots (B) of *SLC4A11* expression in patients with or without lymphatic invasion.

Using data from TCGA-OV, we compared *SLC4A11* expression in ovarian cancer patients with or without lymphatic invasion. Results revealed that the cases with lymphatic invasion (N = 133) had significantly higher *SLC4A11* expression (*p* = 0.0069) than those without lymphatic invasion (N = 77) (Fig 2A and 2B).

### High *SLC4A11* expression is an independent predictor of poor OS in ovarian cancer patients

**Fig 3:**
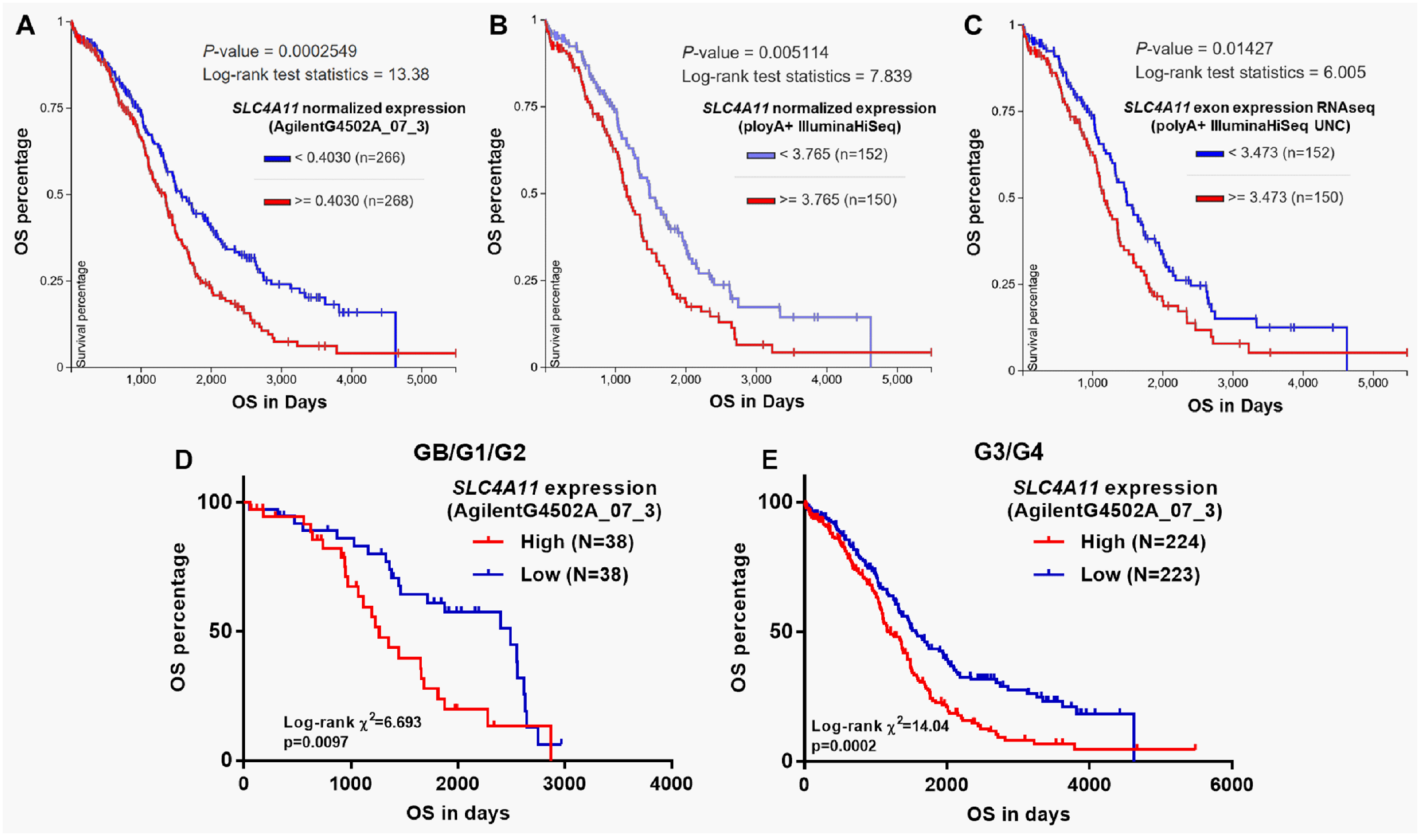
High *SLC4A11* expression is associated with poor survivals in ovarian cancer patients. **A-E**. Kaplan-Meier curves of OS in ovarian cancer patients with high and low *SLC4A11* expression. Data were obtained from TCGA-OV. *SLC4A11* expression was quantified by RNAseq (AgilentG4502A_07_3 (A) and IlluminaHiSeq respectively (B)) and exon RNAseq (polyA+ IlluminaHiSeq) (C). Kaplan-Meier curves of OS in GB/G1/G2 patients (D) and in G3/G4 patients (E).

The association between clinicopathological features and *SLC4A11* expression in patients with primary serous ovarian cancer was summarized in Table 1. High *SLC4A11* expression was significantly associated with lower grade tumors (GB/G1/G2) (*p* = 0.038), and a larger proportion of lymphatic invasion (*p* = 0.027) and deceased cases (*p* = 0.036) (Table 1). In addition, the high *SLC4A11* expression group also had a higher ratio of tumor residual disease at the margin level of significance (*p* = 0.050) (Table 1). However, no significant difference was observed in recurrence rate between the two groups (Table 1). Then, Kaplan-Meier survival analysis was performed between the high and low *SLC4A11* expression groups in TCGA-OV. In the database, *SLC4A11* expression was quantified by RNAseq (AgilentG4502A_07_3 and IlluminaHiSeq respectively) and exon RNAseq (polyA+ IlluminaHiSeq). Among 540 patients with *SLC4A11* measured by AgilentG4502A_07_3, 534 patients had OS data. Both IlluminaHiSeq and RNAseq data included 302 patients with OS data (Fig 2A–2C). Log-rank test showed that in all measurements, high *SLC4A11* expression was consistently associated with worse OS compared with low *SLC4A11* expression (*p* = 0.00025, 0.0051 and 0.014 respectively, Fig 2A–2C). Recent studies suggest that GB/G1/G2 carcinoma belong to type I, while G3/G4 carcinomas belong to type II tumors, which have significant clinicopathologic and molecular differences [20, 21]. We then performed subgroup analysis in GB/G1/G2 and G3/G4 tumors respectively. In both groups, we found that high *SLC4A11* expression was associated with unfavorable OS (Fig 3D and 3E). In univariate analysis, high *SLC4A11* expression was associated with poor prognosis in terms of OS in both GB/G1/G2 and G3/G4 tumors (*p* = 0.011 and *p*<0.001 respectively) (Table 2). However, following multivariate analysis only confirmed the independent prognostic value of high *SLC4A11* expression in G3/G4 tumors (HR = 1.416, 95%CI: 1.098–1.824, *p* = 0.007; Table 2), but not in GB/G1/G2 tumors (HR = 1.832, 95%CI: 0.987–3.402, *p* = 0.055; Table 2). Due to insufficient data of recurrence, we only assessed the association between *SLC4A11* expression and RFS in patients with G3/G4 diseases. No independent prognostic value of *SLC4A11* expression was observed in terms of RFS (Table 2).

**Table 1:** Demographic and clinicopathological parameters of patients with primary ovarian cancer in TCGA-OV.

Parameters		*SLC4A11* expression RNAseq		χ^2^	*p* Value
		High (N = 270)	Low (N = 270)		
**Age (Mean ± SD)**	≥60	131	122	0.54	0.46
	<60	139	147		
	Null	0	1		
**Grade**	GB/G1/G2	46	30	4.30	0.038
	G3/G4	215	236		
	GX + Null	9	4		
**Clinical stage**	I/II	18	24	0.93	0.33
	III/IV	249	243		
	Null	3	3		
**Venous invasion**	No	30	37	2.05	0.15
	Yes	48	37		
	Null	192	196		
**Lymphatic invasion**	No	33	44	4.88	0.027
	Yes	78	55		
	Null	159	171		
**Tumor residual disease**	No	44	62	3.83	0.050
	Yes	194	177		
	Null	32	31		
**Recurrence status**	No	5	10	0.24	0.62
	Yes	21	31		
	Null	244	229		
**Living Status**	Living	99	122	4.38	0.036
	Dead	169	144		
	Null	2	4		

GB: Border line malignancy; G1: Well differentiated; G2: Moderately differentiated; G3-G4: Poorly differentiated or Undifferentiated; GX: Grade cannot be assessed. Null: no data.

**Table 2:** Univariate and multivariate analyses of OS/RFS in patients with primary ovarian cancer in TCGA-OV.

Parameters	Univariate analysis				Multivariate analysis			
	*p*	HR	95%CI (lower/upper)		*p*	HR	95%CI (lower/upper)	
**OS (GB/G1/G2)** **Age** ≥60 vs.< 60	0.011	2.171	1.192	3.956	0.999	1.00	0.549	1.824
**Clinical stage** III/IV vs. I/II	0.018	5.557	1.338	23.07	0.0367	4.63	1.099	19.504
**Venous invasion** No vs. Yes	0.112	0.392	0.123	1.244				
**Lymphatic invasion** No vs. Yes	0.999	1.001	0.27	3.712				
**Tumor residual disease** No vs. Yes	0.208	1.693	0.746	3.841				
***SLC4A11* expression** High vs. Low	0.011	2.171	1.192	3.956	0.055	1.832	0.987	3.402
**OS (G3/G4)** **Age** ≥60 vs.< 60	<0.001	1.616	1.266	2.062	0.001	1.516	1.187	1.937
**Clinical stage** III/IV vs. I/II	0.123	1.743	0.861	3.529				
**Venous invasion** No vs. Yes	0.533	1.2	0.676	2.132				
**Lymphatic invasion** No vs. Yes	0.055	0.613	0.373	1.01	0.563	0.859	0.514	1.437
**Tumor residual disease** No vs. Yes	<0.001	2.46	1.647	3.675	<0.001	2.252	1.497	3.39
***SLC4A11* expression** High vs. Low	<0.001	1.595	1.247	2.04	0.007	1.416	1.098	1.824
**RFS (G3/G4)** **Age** ≥60 vs.< 60	0.3004	1.366	0.757	2.465				
**Clinical stage** III/IV vs. I/II	0.146	0.22	0.029	1.692				
**Tumor residual disease** No vs. Yes	0.017	2.421	1.175	4.989	0.041	2.167	1.031	4.553
***SLC4A11* expression** High vs. Low	0.052	1.769	0.996	3.14	0.179	1.511	0.828	2.757

GB: Border line malignancy; G1: Well differentiated; G2: Moderately differentiated; G3-G4: Poorly differentiated or Undifferentiated. OS: overall survival; RFS: recurrence-free survival.

### *SLC4A11* expression is regulated by DNA amplification and methylation in ovarian cancer

**Fig 4:**
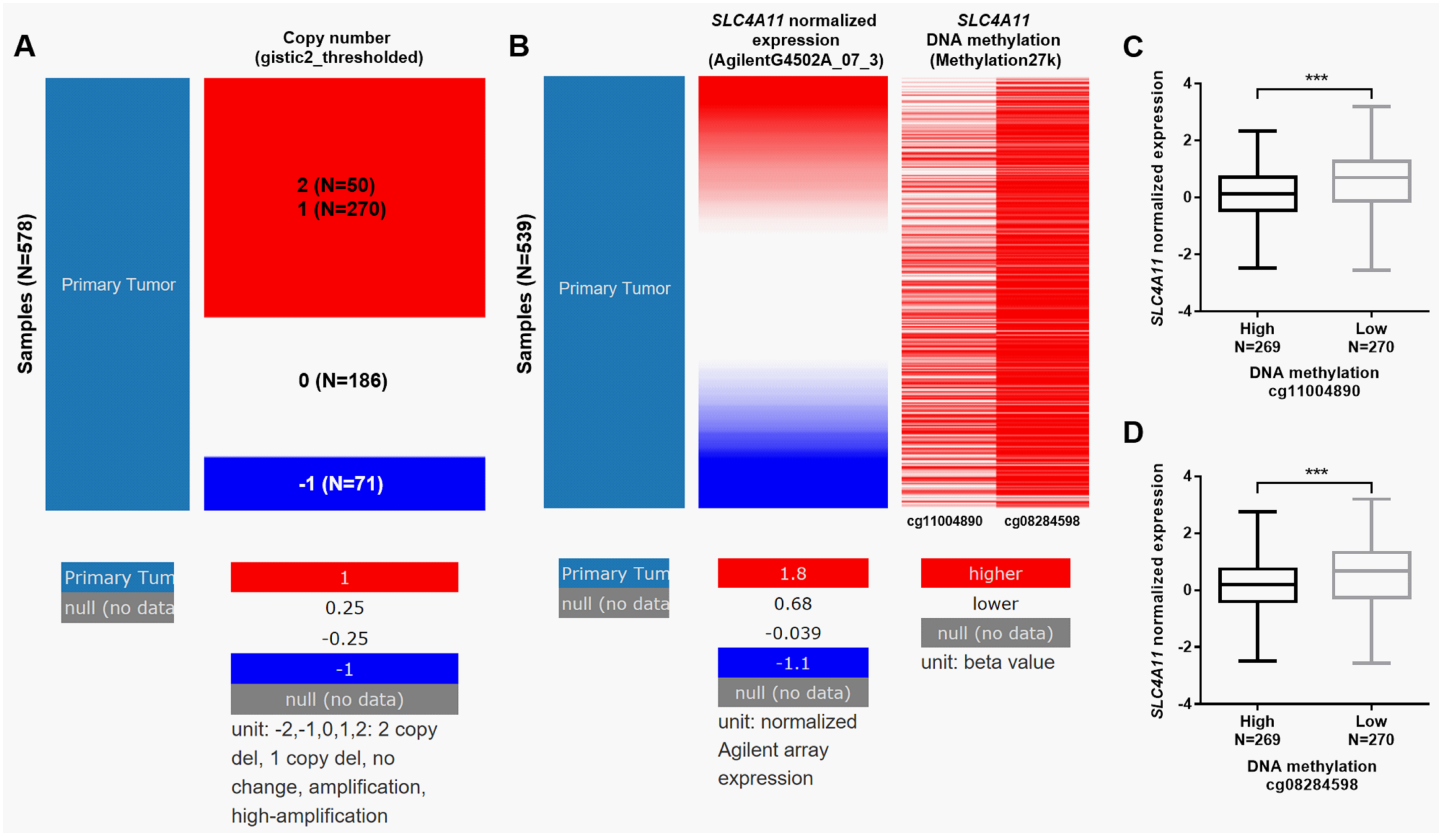
*SLC4A11* expression is regulated by DNA amplification and methylation in ovarian cancer. **A**. Heat map of *SLC4A11* copy number changes in TCGA-OV. -1: deletion; 0: no change; 1: amplification; 2: high-amplification. **B-D**. Heat map (B) and box plots (C-D) of *SLC4A11* expression and DNA methylation in TCGA-OV. Patients were divided into high and low methylation groups according to the median value of the two probes (cg11004890 and cg08284598) respectively.

Via analyzing the deep sequencing data in TCGA-OV, we further explored the mechanisms of *SLC4A11* dysregulation. Among 578 patients with copy number measured, 320 (55.4%) cases had DNA amplification (Fig 4A). By comparing *SLC4A11* expression and its DNA methylation status, we also confirmed that the high methylation group had a significantly lower level of *SLC4A11* expression (Fig 4B–4D).

## Discussion

As a Na^+^-coupled base transporter capable of Na^+^-H^+^ exchange in mammalian cells, *SLC4A11* mutation is associated with a series of corneal endothelial diseases characterized by dysfunction of the endothelial cells in the inner surface of the cornea [22–24]. These diseases finally result in the apoptosis of corneal endothelial cells, and subsequent edema and impaired vision [25]. In fact, the normal corneal endothelial function is dependent on the balanced regulation of bicarbonate concertation and pH_i_/pH_e_. Based on these findings, we infer that the putative bicarbonate transport or Na^+^-H^+^ exchange activity of SLC4A11 is essential for maintaining normal cellular physiological functions.

In this study, we found that *SLC4A11* expression is significantly upregulated in ovarian cancer tissues than in normal tissues. The alkaline intracellular environment of cancer cells is usually associated with the upregulation of the expression and/or activity of acid extruders or downregulation of the expression and/or activity of acid loading transporters [1]. Given the pHi regulatory activity demonstrated in previous studies [10, 26], SLC4A11 naturally owns a strong cellular buffering capacity that enables the highly glycolytic cancer cells to remove lactic acid rapidly. Its upregulation can be considered as an adaptive response of the cancer cells to metabolic changes. In addition, upregulation of the acid extruders leads to extracellular acidification, thereby generating a favorable environment for tumor invasion and metastasis [5, 6]. In this study, we observed that the patients with metastasis had significantly higher *SLC4A11* expression than their counterparts without metastasis. Therefore, we infer that *SLC4A11* upregulation is an important mechanism leading to malignant cellular behaviors in ovarian cancer. By comparing the clinicopathological features between high and low *SLC4A11* expression groups, we found that the high *SLC4A11* expression group had a significantly higher ratio of lymphatic invasion and deceased cases. However, we also observed that the high *SLC4A11* expression group had a substantially larger proportion of low-grade tumors (GB/G1/G2) (46/261, 17.6% *vs*. 30/261, 11.3%) compared with the low *SLC4A11* group. Actually, G1/G2 serous ovarian carcinomas belong to type I tumors, while G3/G4 serous ovarian carcinomas belong to type II tumors, which have different molecular and biological profiles [20, 21]. We hypothesized that the difference in tumor grade might be related to the genetic difference between type I and type II tumors. However, more studies are required to confirm this hypothesis.

Currently, a series of predictors such as clinical stage, CA125 levels, age, response to chemotherapy and residual disease after debulking surgery are used to predict prognosis of ovarian cancer patients. However, there is still some discrepancy in the prognosis of similar tumors characterized by these clinical variables [27]. Ovarian cancers actually are a heterogeneous group of neoplasias derived from the ovarian surface epithelium, inclusion cysts, or the fallopian tube [28, 29]. The differences at the molecular level may be important sources of the variations. Therefore, it is necessary to explore other molecular parameters for better prediction of prognosis. In this study, based on the survival data of patients in TCGA-OV, we found that high *SLC4A11* expression was robustly associated with poor OS. Following univariate and multivariate analysis confirmed that high *SLC4A11* expression was an independent prognostic factor for poor OS in G3/G4 tumors. These findings suggest that *SLC4A11* might be a potential clinical marker in this group of patients. Although the association between *SLC4A11* expression and OS in GB/G1/G2 patients was not significant, it showed a trend toward significance (*p* = 0.055). Considering the small number of GB/G1/G2 patients included in this study (N = 76), it is meaningful to further explore its prognostic value with a large sample base in the future.

*SLC4A11* locates in chromosome 20p in the human genome, a region with a relatively high frequency of genetic amplification in ovarian cancer [30, 31]. By using deep sequencing data in TCGA-OV, we found that 320 out of 578 ovarian cancer cases had *SLC4A11* amplification. Therefore, DNA amplification is one important mechanism of upregulated *SLC4A11* in ovarian cancer. Epigenetic regulation, such as methylation and histone modification are also important mechanisms of dysregulated genes in ovarian cancer [32, 33]. Thus, we also investigated whether methylation status influences *SLC4A11* expression. By using the results from Illumina 27k methylation array, we found that the group with high DNA methylation had significantly lower *SLC4A11* expression, suggesting that epigenetic alterations also contribute to *SLC4A11* upregulation in ovarian cancer.

## Conclusion

Based on findings above, we infer that high *SLC4A11* expression is an independent predictor for poor OS in grade 3/4 serous ovarian cancer. Both DNA amplification and hypomethylation contribute to its upregulation in ovarian cancer.

## Supporting information

S1 Table Original data downloaded from the UCSC Xena Browser. (XLSX)
